# Spark Plasma Sintered Soft Magnetic Composite Based on Fe-Si-Al Surface Oxidized Powders

**DOI:** 10.3390/ma15227875

**Published:** 2022-11-08

**Authors:** Traian Florin Marinca, Bogdan Viorel Neamțu, Florin Popa, Amalia Mesaroș, Ionel Chicinaș

**Affiliations:** 1 Materials Science and Engineering Department, Technical University of Cluj-Napoca, 400641 Cluj-Napoca, Romania; 2 Physics and Chemistry Department, Center for Superconductivity, Spintronics and Surface Science, Technical University of Cluj-Napoca, 400114 Cluj-Napoca, Romania

**Keywords:** spark plasma sintering, Sendust, soft magnetic composite, surface oxidized powder, reaction sintering

## Abstract

Soft magnetic composites (SMCs) need a stable matrix to apply heat treatments for enhancing their magnetic characteristics. A stable matrix can be offered by alumina, but the densification of the ferromagnetic particles covered by this oxide (by sintering) can be very difficult. This paper proposes a feasible synthesis route for obtaining alumina matrix SMCs. An Fe-Si-Al alloy with nominal composition Fe85Si9Al6 was obtained by mechanical alloying of elemental Fe, Si, and Al powders, and further, the as-milled powders were superficially oxidized by immersion in HCl solution. The oxide layer was composed of iron, silicon, and aluminum oxides, as the Fourier-transform infrared spectroscopy technique revealed. The Fe-Si-Al@oxide powder was densified by the spark plasma sintering technique—SPS. Upon sintering, a continuous matrix of oxide (mainly alumina) was formed by the reaction of the Fe-Si-Al powder coreswith their oxide layer. The main part of the composite compacts after sintering consisted of an Fe_3_Si-ordered phase dispersed in an oxide matrix. The DC and AC tests of magnetic composite compacts showed that upon increasing the sintering temperature, the density, magnetic induction, and magnetic permeability increased. The initial magnetic permeability was constant in the entire range of testing frequencies and the magnetic losses increased linearly. The stability of the magnetic characteristics in frequency is promising for developing further such types of magnetic composite.

## 1. Introduction

More and more complex technologies need high-performance magnetic materials. Among these high-performance materials, soft magnetic composites (SMC) are a major element of focus. Soft magnetic composites have been the main subject of research for decades, due to their promising characteristics such as high electrical resistivity, good magnetic permeability and level of induction, and the possibility of their being easily shaped into various 3D designs [1,2,3,4]. To obtain a soft magnetic composite, a ferromagnetic part must be isolated by a layer that can be organic or inorganic and, further, the powder must be densified. Ferromagnetic parts based on Fe and Fe-based alloys are principal candidates [1] and a large range of SMCs containing Fe/Fe-based alloys have previously been developed, such as Fe/resin/Ni-Zn ferrite nanofibers [5], Fe@SiO_2_/ferrite [6], Fe/Silicate glass [7], Fe/nano-ZnO/boron oxide [8], Fe/polymer [9], and FeSiAl/Al_2_O_3_–MnO–Al_2_O_3_ [10]. The method of covering and the materials that cover the ferromagnetic part are basic aspects of SMC [1,6,10]. Besides these, another important aspect is the densification technique; a good densification technique that allows the acquisition of very dense compacts will provide proper magnetic characteristics. Sendust-type alloys are Fe-based alloys, containing Si and Al, in addition to Fe. Masumoto and Yamamoto from the Tohoku Imperial University of Sendai, Japan, developed these Fe-Si-Al alloys several decades ago. Sendust was invented as an alternative to permalloy. This iron alloy, with Si content of 6–11 wt% and Al content of 4–8 wt% shows excellent soft magnetic properties (low power losses, good level of magnetic induction, high magnetic permeability, and low coercivity) and is low cost [11,12]. Several soft magnetic composites based on Sendust-type powder have been obtained to date [10,13,14,15,16,17,18]. Generally, for SMC compact preparation, the powder, covered by various types of insulating layers, is pressed into the desired shape and post-densifying annealing is applied [4,5,6]. Among the densification techniques, spark plasma sintering is a good candidate, due to its interesting features such as low sintering temperature, high heating rates, and short dwelling time. These allow for the densification of a large variety of materials, besides the classical ones, and of great interest currently is the densification of composites. The low sintering temperatures allow for the sintering of composite powders that if sintered by the classical route would react with each other [19,20]. Of interest is the use of the SPS technique to promote the reaction between phases and to control the reaction, in order to obtain a dense composite material [21]. This technique was successfully used to obtain soft magnetic composite compacts by reaction-sintering both Fe-based [15] and Ni-based materials [22]. An interesting and useful approach is the densification by SPS of the mechanically-milled powder. The powder can be activated by the mechanical milling process, and this represents a useful aspect both from sintering and also from the covering of the particles with an insulating layer [23,24,25,26].

The purpose of this work is to provide a simple way of preparing soft magnetic sintered compacts, based on an iron alloy that allows very good thermal stability and has high electrical resistivity offered by its alumina matrix. In this research, Fe-Si-Al particles are covered, by a simple chemical method, with an oxide layer and, further, these composite particles (with a core-shell structure) are sintered by spark plasma sintering. Our approach is to use Fe-Si-Al alloy in a nanocrystalline state, obtained via a synthesis route, mechanosynthesis–mechanical alloying and milling, which involves severe plastic deformation, leading to a powder with multiple structural defects [27] and high energy amount inside generated by internal stresses, that will promote the sintering and the reactions during SPS. The Fe-Si-Al alloy reacts with its layer of oxide upon sintering, to form an alumina-based ceramic matrix of high electrical resistivity, in which are embedded Fe-Si ferromagnetic zones that ensure good magnetic properties.

## 2. Materials and Methods

Elemental powders of Fe (NC100.24 produced by Höganäs AB, Höganäs, Sweden), Si (Alpha Aesar, Kandel, Germany), and Al (Alpha Aesar, Kandel, Germany) have been used for Fe85Si9Al6 (wt%) alloy synthesis. The elemental powders have been milled for up to 15 h in a planetary ball mill manufactured by Fritsch, model Pulverisette 6. The mechanical alloying process has been carried out in a high-purity argon atmosphere and the process has been stopped after 2, 4, 6, 8, and 10 h for sampling. The rotational speed has been established at 400 rpm. The vial (500 mL volume) and balls (14 mm diameter and 12 g each) are made of hardened steel. The ball-to-powder ratio was 8:1. After the synthesis of the Fe-Si-Al alloy, as a single phase, the powders have been immersed in an HCl solution to create an oxide layer on the surface. By this simple technique, Fe-Si-Al@oxide core-shell type particles have been obtained. The acidic solution was obtained by dilution of 1 mL of hydrochloric acid, HCl, (37%) into 200 mL of distilled water. The immersion time was 12 h. After the immersion, the samples have been washed three times with distilled water. The samples have been dried in air at room temperature. The oxidized powders have been sintered by homemade spark plasma sintering equipment, at sintering temperatures of 800 and 900 °C with a dwelling time of 10 min. The installation works at 24 V and 3.75 kA and uses graphite die and punches. The samples have not been cold pressed before sintering. A pressure of 30 MPa was constantly applied during the sintering procedure. The heating rate was about 5 °C/s and the cooling of the sample was undertaken in about 30 min, in the oven. The sintering process has been performed in an argon atmosphere. The samples have been prepared, from the metallographic point of view, by grinding with silicon carbide foils and polishing with fine alumina solution on a polishing-cloth disc. The density of the sintered compacts has been measured by a Precisa XT220A balance (Precisa Gravimetrics AG, Dietikon, Switzerland), equipped with a density determination module, based on the Archimedes method. Fourier-transform infrared spectroscopy (FTIR) was carried out for verifying the formation of an oxide layer on the surface of the particles. The powders have been diluted in KBr powder and tested by a Tensor 27 Bruker FTIR spectrophotometer (Karlsruhe, Germany). The X-ray diffractions have been carried out by an Inel Equinox 3000 apparatus (INEL, Artenay, France), which works in reflection mode using Co radiation in a 2-theta range of 20–110 degrees. The crystallite size (D) calculations have been made by Scherrer’s equation: $D=\frac{K\cdot \lambda }{\beta \cdot \cos\theta }$ (*K* is a constant dimensionless shape factor—0.9 value), λ—X-ray wavelength, *β*—diffraction line broadening at half of the maximum intensity (FWHM—Full Width at Half Maximum) and *θ*—Bragg angle [28]. Scanning electron microscopy—SEM was used for powders and compact investigations, using a Jeol-JSM 5600 LV microscope (Tokyo, Japan). Both techniques—SEI: secondary electron image and BEC: backscattered electron contrast—allowed by microscopy, have been used. The local chemical analyses were performed by X-ray microanalysis, using an energy-dispersive X-ray (EDX) detector, ULTIMMAX65 model, Oxford Instruments (High Wycombe, UK), which equipped the scanning electron microscope (Aztec software. Version 5.0). The electrical resistivity of the compacts has been determined using a four-point homemade installation. The DC and AC characteristics of the sintered samples have been investigated by computer-controlled hysteresisgraph Magnet-Physik Dr. Steingroever GmbH, Remagraph–Remacomp C–705 model, (Cologne, Germany).

## 3. Results and Discussion

### 3.1. Fe-Si-Al Powder Obtained by Mechanical Alloying

#### 3.1.1. Structural Investigation by X-ray Diffraction

**Figure 1:**
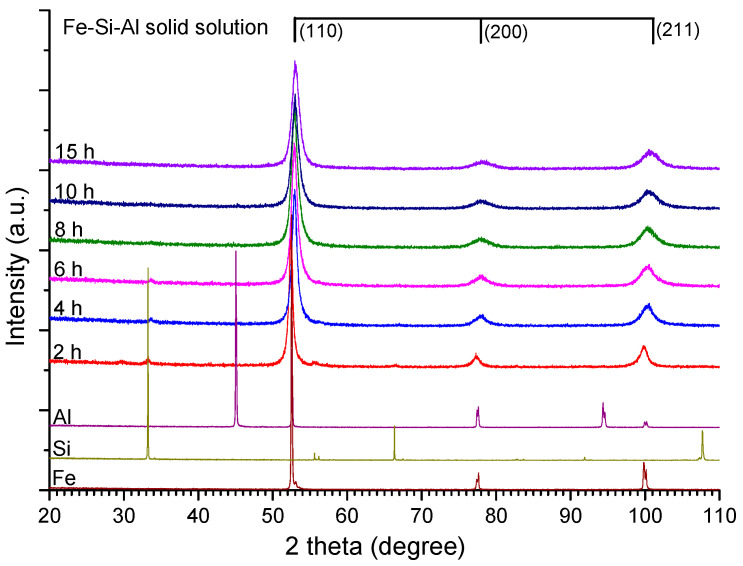
X-ray diffraction patterns of the Fe-Si-Al mechanically alloyed powder. The Fe, Si, and Al patterns are given for comparison.

The X-ray diffraction patterns of the Fe-Si-Al milled samples are presented in Figure 1. In the same figure are presented, for comparison, the X-ray diffraction patterns of the Fe, Al, and Si powders used in the mechanical milling process. The alloy forms progressively, up to 10 h of milling. At this milling time in the diffraction pattern are visible only the maxima of a BCC single-phase, Fe-based. Based on the XRD technique at this point, the Fe-Si-Al solid solution is formed as a single phase. The Al maxima are not visible in the diffraction patterns from 2 h of milling and those of Si are not visible from 10 h. The mechanical alloying process has been continued up to 15 h of milling, to be certain that the solid solution is homogeneous at the atomic level, considering that the XRD techniques can indicate a single phase, but some nanoclusters rich in some alloying elements can exist (the detection limit of the XRD technique is about 3–5%). The mean crystallite size after 15 h of MA is around 10 nm. The Fe and Fe-Si-Al have been indexed according to JCPDS file no. 06-0696, Si to JCPDS file no. 27-1402 and Al to JCPDS file no. 04-0787.

#### 3.1.2. Powder Morphology Investigation by Scanning Electron Microscopy

**Figure 2:**
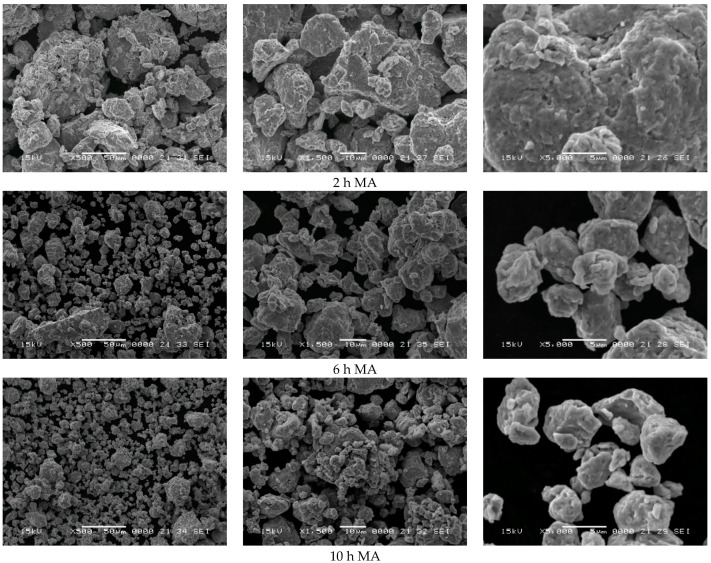

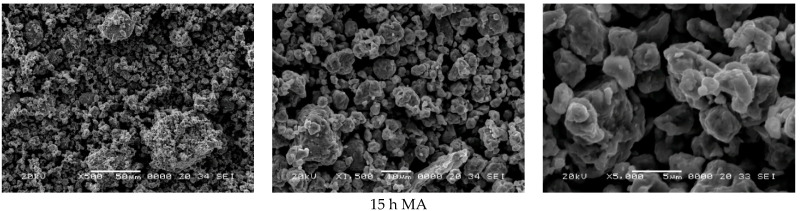
Scanning electron microscopy images in secondary electron mode of the sample milled for 2, 6, 10, and 15 h.

The scanning electron microscopy images obtained in the secondary electron signal of the mechanically-alloyed powders are shown in Figure 2. One aspect worthy of note is the particle size reduction upon increasing the milling time. At lower milling times, the size of the particles is large, and particles of tens of micrometers can be identified; even particles larger than 100 μm are visible in the recorded images. This is expected, since the iron particles at the beginning of milling have a mean size of 100 μm. The progressive incorporation of Al and Si into the Fe structure leads to the fragilization of the structure and, thus, by milling, the refinement of the particles occurs. After 15 h of mechanical milling, the particle size is rarely more than 10 μm. At high magnification, it can be noticed that the larger particles are formed by the agglomeration/cold welding of small particles of a few micrometers.

#### 3.1.3. Local Chemical Homogeneity–Energy Dispersive X-ray Spectroscopy

**Figure 3:**
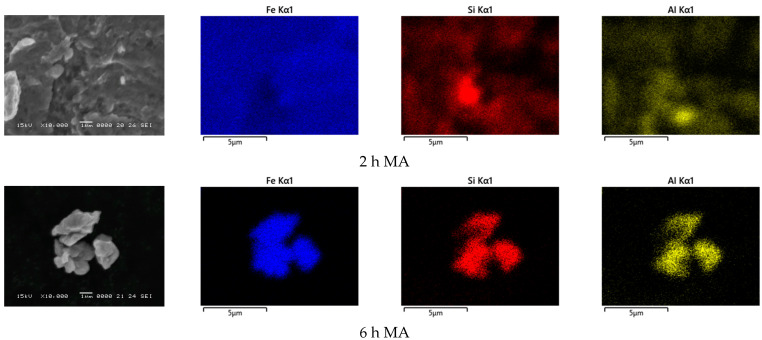

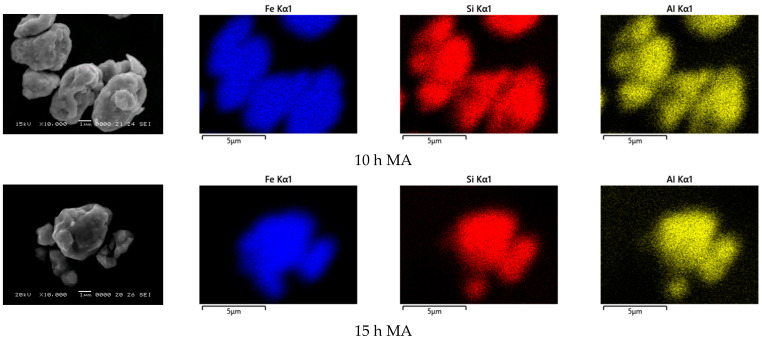
Chemical elements’ distribution maps, obtained by EDX analysis, of the samples milled for 2, 6, 10, and 15 h (10,000× magnification.)

The Fe, Si, and Al chemical elements’ distribution maps, taken by EDX analysis, of the samples milled for 2, 6, 10, and 15 h, are shown in Figure 3. After 2 h of mechanical milling, it was observed that the Fe was already uniformly distributed in the analyzed area, acting like a matrix. Si and Al atoms are present in the entire investigated zone but there are areas where are remarked concentrations of these elements This shows that after 2 h of milling, the homogenization of the chemical elements is good, at the micrometer level. In the zones where high concentrations of Si were observed, they have a mean diameter of 1–3 μm. The EDX investigations are in good agreement with the XRD investigations, regarding the Si. The XRD showed the presence of a Si-based phase in the powder at this milling time. The XRD did not show the presence of an aluminum-based phase, due to the low crystallinity of this phase, or its low quantity, which cannot be detected by XRD. The Al-concentrated areas are assigned to an Al-based phase, which is present in a lower amount and has very low crystallinity. Upon increasing the alloying time to 6 h, a much more uniform distribution of the chemical elements was noted. At this magnification level, no areas with increased concentrations of Si and/or Al were observed. The XRD revealed the presence of a Si-based phase for this milling time. Due to the existence of this phase at the nanoscale, it was not evidenced by EDX, considering the magnification level. In the EDX investigations for the samples milled for 10 and 15 h, the elemental distribution maps show uniform distribution. 

### 3.2. Fe-Al-Si@oxide Core-Shell Powder

**Figure 4:**
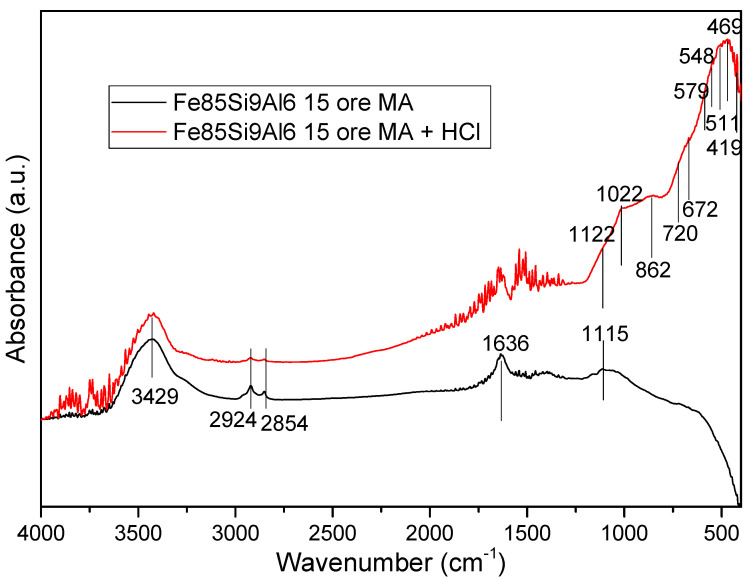
FTIR spectra of as-milled Fe-Si-Al alloy, before and after immersion in HCl solution.

The FTIR spectra of the (as-milled) Fe-Si-Al alloy, after 15 h of mechanical milling, and after 15 h of milling and further immersed in HCl solution, are presented in Figure 4. Some vibrational bands, typical for samples manipulated in air, are present: 3429 and 1636 cm^−^^1^—O–H group bending and O–H symmetrical stretching of H_2_O. The bands at 2924 and 2854 cm^−^^1^ are associated with the O–H of absorbed water on the surface of the powder. The sample after milling presents a band at about 1115 cm^−^^1^ and this band is assigned to the O–H bending mode of H_2_O absorption on the Fe-Si-Al particles. The manipulation of the samples in air also led to the appearance of these bands [28,29,30].

After the immersion of the Fe-Si-Al particles in HCl, other new bands were remarked: 1122, 1022, 862, 720, 672, 579, 548, 511, 469, and 411 cm^−1^. The band at 1122 cm^-1^ is similar to the band at 1115 cm^-1^ in the sample without immersion in HCl, presenting a minor shift. The band at about 1022 cm^-1^ is assigned to the Si-O asymmetric stretching vibration of the SiO_2_ that is formed on the powder surface [31,32]. This indicates the formation of the SiO_2_ compound. The other important band of SiO_2_ is at about 511 cm^−1^ (bending vibration mode in O-Si-O) and is partially covered by other bands close to this value. The bands centered at 548 and 720 cm^−1^ are assigned to Al-O bands from Al_2_O_3_ to tetrahedral (Al-O_4_) and octahedral (Al-O_6_) groups [33,34,35]. The bands of Me-O (Al-O and Fe-O) are all at around 500 cm^−1^ and therefore they are superposed, entirely or partially, giving a large band. The bands identified at 672 and 469 cm^−1^ are assigned to Fe-O vibrational mode in ferric oxide, Fe_2_O_3_. The band at about 411 cm^-1^ is attributed to the Fe-O vibrational mode of magnetite, Fe_3_O_4_. The Fe_3_O_4_ has another important band at about 570 cm^−1^ that is partially overlapped with other bands. The band at 862 cm^-1^ is assigned to the Fe-OH groups’ band-bending vibration. Fe-OH bonds are formed by water molecules’ absorption on the surface of fine particles [32]. The presence of the Si-O, Al-O, and Fe-O vibrational bands, on the FTIR spectrum of the sample immersed in HCl solution, and the absence of those bands on the as-milled sample, indicates the presence of the oxide layer on the surface of the Fe-Si-Al powders and the formation of the Fe-Si-Al@oxide core-shell type particles. The FTIR investigations suggest the presence of Al_2_O_3_, SiO_2_, Fe_2_O_3,_ and Fe_3_O_4_.

### 3.3. Spark Plasma Sintered Composite Compacts

#### 3.3.1. Compacts’ Microstructure and Density

**Figure 5:**
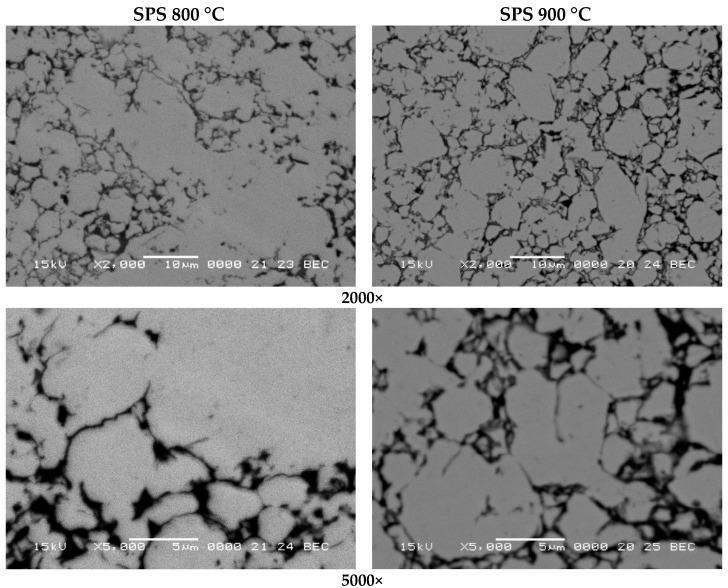
Figure **5.** Scanning electron microscopy micrographs, obtained by backscattering electrons’ signal at 2000× and 5000×, of the samples densified at 800 and 900 °C by spark plasma sintering.

Figure 5 presents the scanning electron microscopy micrographs, obtained by backscattering electrons signal (BEC) for the samples, densified at 800 and 900 °C by spark plasma sintering. As is well known, the BEC signal of SEM provides the information about different phases, and the image provided by this signal offers information about the sample’s phase composition. This is a very suggestive approach to investigating composite samples. Our sample, investigated by BEC signal, shows mainly two types of phase: the first type is given by lighter zones that are not continuous, and the second type is given by darker zones that present like a continuous matrix [22]. The darker zone looks like a continuous network, similar to the iron carbide one in hypoeutectoid steels. The lighter zones are the main part of the microstructure, and are associated with some iron-based phase/phases, considering that the main phase of the particles used for compact synthesis is Fe-SI-Al solid solution. The darker network is assigned mainly with oxides. Also, in that network, pores are assigned to be present. Upon sintering, the oxides can be similar to those that covered the Fe-Si-Al powder, or others resulting from the reaction of Fe-Si-Al solid solution with the oxide that covered the Fe-Si-Al particles. The sample sintered at 800 °C is less dense, when compared to the one sintered at 900 °C with 8%. The density of the sample sintered at 800 °C is 5.19 g/cm^3^ and the density of the other compact is 5.61 g/cm^3^. The better density is given by higher sintering temperature (the dwell time is the same) [20]. At 900 °C the sintering process is more favorable to the sample densification and is linked to the atoms’ enhanced diffusion.

#### 3.3.2. Chemical Elements’ Distribution

**Figure 6:**
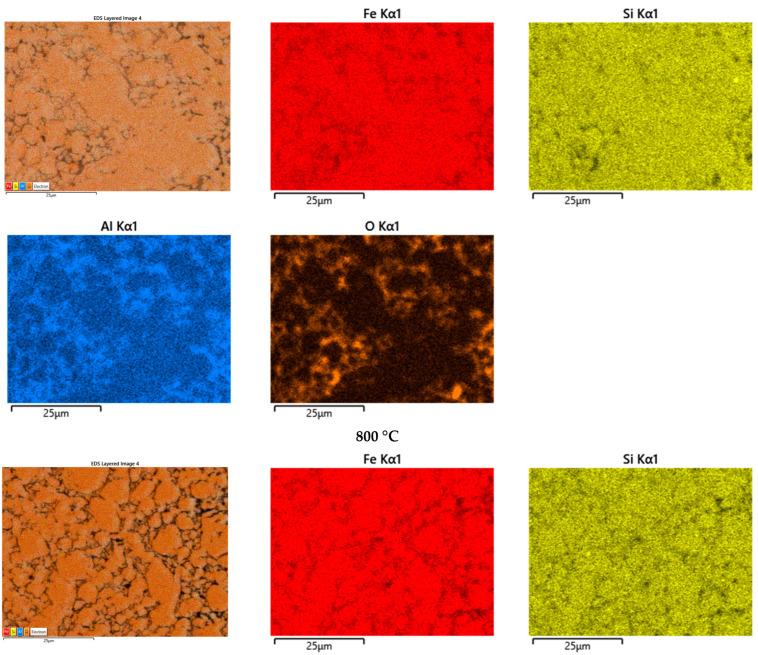

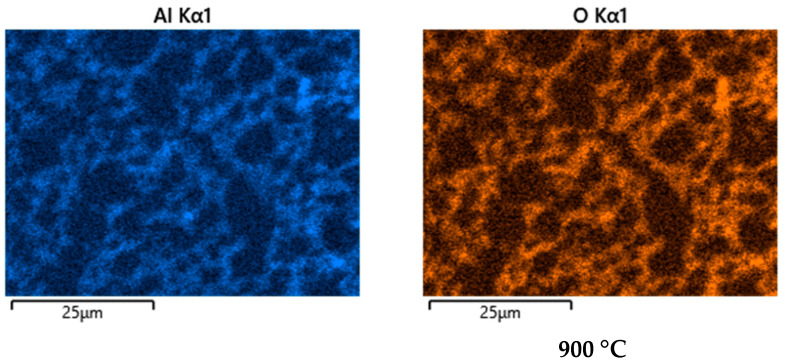
Chemical elements’ distribution maps of the compacts sintered at 800 and 900 °C.

To check the distribution of the elements in the sintered samples, elements’ distribution maps were recorded by energy-dispersive X-ray spectrometry. The EDX investigations confirmed the assignation of the zones that was undertaken upon analyzing the scanning electron microscopy micrographs. Figure 6 depicts the chemical elements’ distribution maps for the compacts sintered at 800 and 900 °C. It can be remarked, in the case of the compact sintered at 800 °C, that the Fe and Si atoms have a similar distribution in the analyzed zones and coincide with the lighter zones identified by SEM. This is the main part of the composite and is predominantly metallic zones. Oxygen atoms surrounded the zones where the Fe and Si atoms were distributed. In the areas where the oxygen atoms were distributed, an agglomeration of Al atoms was noted. This suggests that some aluminum oxide was formed, mainly by reaction during spark plasma sintering. The Al atoms, being more reactive with oxygen, compared to the others present in the sample, diffuse to the surface of the particles and react with the other oxide that has been formed upon the immersion of the Fe-Si-Al particles in HCl solution. The diffusion does not lead to the complete Al atoms’ depopulation in the metallic zones, because they are also present in those zones, but in a smaller amount. Upon increasing the sintering temperature to 900 °C the Fe, Si, and O have similar distributions, as in the case of compacts sintered at a lower temperature. The Al atoms’ distribution is different: it is less present in the zones where Fe and Si atoms are present, when compared with those observed at lower sintered temperatures. This indicates a better diffusion of these atoms to the surface of the Fe-Si-Al particles, and probably aids promotion of the reaction between the core and oxides layer. The reaction of Al atoms with the oxides that exist at the surface of the particles (iron and silicon oxide) results in Fe and Si atoms. These atoms diffuse into the core of the particles upon sintering. The diffusion of Si and Fe atoms into the cores of particles (now lighter zone, the metallic ones) leads to a change in these zones’ stoichiometry. The considered main reactions in the metallic-oxidic interface, upon sintering, are:

(1) $$3{\mathrm{SiO}}_{2}+4\mathrm{Al}\to 2{\mathrm{Al}}_{2}O_{3}+3\mathrm{Si}$$

(2) $${\mathrm{Fe}}_{2}O_{3}+2\mathrm{Al}\to {\mathrm{Al}}_{2}O_{3}+2\mathrm{Fe}$$

(3) $$3{\mathrm{Fe}}_{3}O_{4}+8\mathrm{Al}\to 4{\mathrm{Al}}_{2}O_{3}+9\mathrm{Fe}$$

#### 3.3.3. Phases in SPSed Compacts

**Figure 7:**
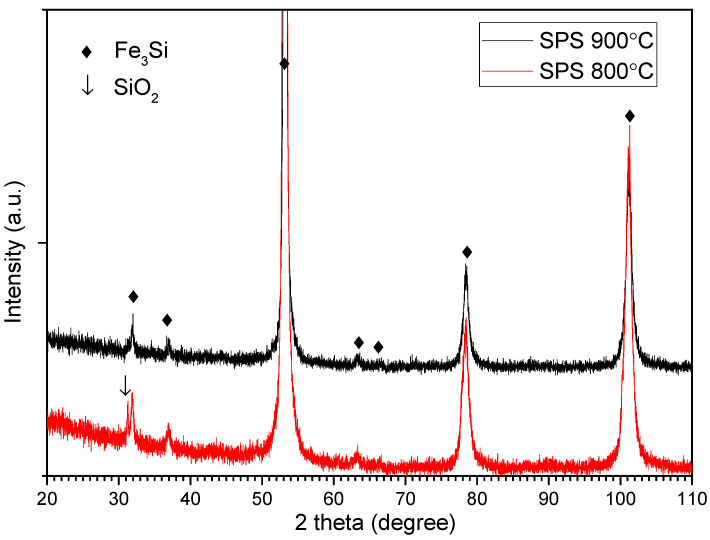
X-ray diffraction patterns of the compacts densified by spark plasma sintering.

The phases that are present in the sintered samples have been checked by the X-ray diffraction technique. The X-ray diffraction patterns of the compacts densified by spark plasma sintering are presented in Figure 7.

Although EDX has revealed some aluminum oxide, by XRD this phase is not obvious in any of the sintered samples. The absence of alumina maxima can be explained by its low crystallinity and also by its distribution in the sintered compacts. It can be assumed that a large part of alumina does not present long-range order. For both sintered samples, the main phase has a structure based on an Fe_3_Si-ordered compound. Upon sintering at both temperatures, the Fe-Si-Al solid solution, by eliminating the aluminum atoms, is ordered. The Fe-Si composition after Al diffusion is in the range of the Fe-Si alloy where the ordering is depicted by the binary phase diagram. In the case of the samples sintered at 800 °C, the most intense maximum of SiO_2_ is present in the diffraction pattern. This phase is present as a shoulder of the Fe_3_Si maxima, at about 32 degrees. Fe_3_Si is an ordered structure with crystal symmetry (Fm-3m) of DO_3_ type [36]. The Fe_3_Si maxima are wide, suggesting that the crystallite size is not at a high value, compared to those of Fe-Si-Al from powder. Indeed, the mean crystallite size is close to the value calculated for powder: 14 nm. In the case of the powder, the mean crystallite size was calculated for samples with mechanically-induced stress and the value may be underestimated.

### 3.4. DC and AC Magnetic Properties of SPSed Toroidal Magnetic Cores. Electrical Resistivity

The DC magnetic characteristics of the toroidal magnetic cores, obtained by sintering using the SPS technique, are given in Table 1, alongside their electrical resistivity and density.

The better magnetic characteristics, from the point of view of magnetic permeability and maximum induction, are given by the toroidal core obtained by sintering at 900 °C. The magnetic core obtained by sintering at 800 °C has a lower coercive field. The better magnetic induction is assigned to a denser zone of atoms with a magnetic moment in the metallic zones. This shows that upon sintering at 900 °C the Al atoms are depopulating the metallic zones from the composite and, thus, leading to more magnetic atoms. By enhancing the reaction between the core of the sintered particles and the shell, the magnetic permeability increases. The better magnetic permeability, upon increasing the sintering temperature, is assigned (besides the changing of composite component phases through reactions upon sintering), to the better density. It is well known that permeability has a strong dependence on material density and porosity [6,37,38]. According to all the data, the coercive field is expected to be lower in the compact with better density if it is kept in mind that they have similar structures and similar phases. Although this is not happening, and the coercive field is larger for the toroid with higher density. The explanation for this is that it should have more pinning centres that will act as a barrier from domain wall motion in the sample sintered at 900 °C [37,38]. This can be linked to some differences between the amount and type of oxides of the matrix that exist between compacts. Additionally, this can be related to the changed interface between the matrix and metallic clusters. Defects in the Fe-based alloy structure may also be expected to be more pronounced in the sample sintered at higher temperature, due to the different chemical composition, given by fewer Al atoms. The electrical resistivity is higher in the case of the compact obtained by sintering at 800 °C. The higher electrical resistivity is assigned mainly to the lower density.

**Figure 8:**
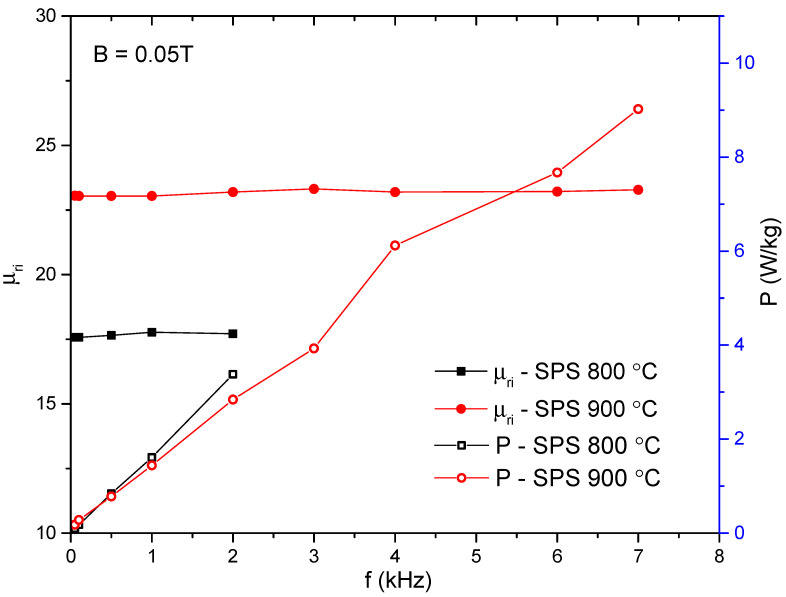
Evolution of magnetic losses and initial magnetic permeability as a function of frequency for toroidal-shaped sintered compacts.

The compacts have also been tested also in the AC magnetization regime, as has already been mentioned. The evolution of magnetic losses, and initial magnetic permeability as a function of frequency for toroidal-shaped sintered compacts, are represented in Figure 8. The initial magnetic permeability is constant in the entire frequency range where the compacts have been tested. This is a very positive aspect; from an application point of view, this is one of the main aspects [1]. The constancy of the permeability is given by the high electrical resistivity (given by the insulating matrix of oxides that covers the particles), which hinders the development of inter-particles eddy currents that can diminish the magnetic permeability [1,39]. It can be observed that the magnetic permeability is better upon increasing the sintering temperature by more than 20%. As has been detailed, the behavior of the permeability in DC is linked to the occurring reactions, to the better density, and to the changing of composite component phases. With regard to the evolution of the magnetic losses versus frequency, it may be remarked that for both toroidal samples the evolution is linear. The evolution of the losses versus frequency operation indicates the limitation of the eddy currents that developed [1,20,39] due to the good insulation of the metallic clusters in the sintered oxide matrix, mainly alumina.

The proposed synthesis procedure can be applied to other Fe-based alloys that incorporate Al atoms, for obtaining soft magnetic composites with high electrical resistivity, which can ensure magnetic cores’ operation at superior frequencies. The alumina matrix ensures an enhanced electrical resistivity, and, at the same time, the alumina–Fe alloy interface is more stable, compared to the cubic spinel ferrite–alloy interface, upon applying heat treatments to improve the magnetic properties. So, the alumina matrix composite is an ideal candidate for high annealing temperatures. Also, if the soft magnetic composites with alumina matrix are compared to polymer matrix soft magnetic composites, similar advantages can be found [1,5,14,17,21,26].

## 4. Conclusions

A simple way to obtain Fe-based soft magnetic composite with oxide matrix was used successfully. Fe-Si-Al powder (Sendust type) was obtained first, by mechanical alloying. The Fe-Si-Al alloy was in a nanocrystalline state, and the powder particles were a few micrometers in size. The alloyed powders were successfully oxidized at the surface by immersion in an HCl solution for 12 h. The FTIR investigations revealed that the Fe-Si-Al powder was covered by a layer of oxides, consisting of a mixture of iron, silicon, and aluminum oxides. The densification of Fe-Si-Al@oxide composite powders was undertaken successfully, using the spark plasma sintering technique. The spark plasma sintering route promotes the rapid reaction between the Fe-Si-Al alloy and its surface oxide layer. Upon sintering, an alumina-matrix is formed by reaction, and the matrix gives a high electrical resistivity to the compacts. The metallic zones are embedded in this alumina matrix. The metallic part is the main part of the sintered composite. The metallic part is given by a Fe3Si-ordered cubic-ty structure. The magnetic permeability is stable for all tested frequencies and the magnetic losses increase linearly upon increasing the frequencies. This clearly shows that the eddy currents are limited/avoided in the tested frequency range. Due to the high resistivity, the operating frequencies could be higher. It is believed that the proposed procedure could be successfully applied to other Fe-based alloy systems containing Al and/or Si, in order to obtain sintered soft magnetic composites.

## Figures and Tables

**Table 1:** DC magnetic characteristics of toroidal magnetic cores and their electrical resistivity and density.

Magnetic Core	Maximum Relative Permeability	Maximum Induction (T)	Coercive Field (A/m)	Electrical Resistivity (Ω·m)	Density (g/cm^3^)
SPS 800 °C	18.6	0.17	173	2.66 × 10^−1^	5.19 ± 0.1%
SPS 900 °C	23.6	0.22	256	3.75 × 10^−3^	5.61 ± 0.1%

## Data Availability

The authors confirm that the data supporting the findings of this study are available within the article.
